# Dual number-based variational data assimilation: Constructing exact tangent linear and adjoint code from nonlinear model evaluations

**DOI:** 10.1371/journal.pone.0223131

**Published:** 2019-10-16

**Authors:** Jann Paul Mattern, Christopher A. Edwards, Christopher N. Hill

**Affiliations:** 1 Ocean Sciences Department, UC Santa Cruz, Santa Cruz, CA, United States of America; 2 Department of Earth, Atmospheric and Planetary Sciences, Massachusetts Institute of Technology, Cambridge, MA, United States of America; University of Michigan, UNITED STATES

## Abstract

Dual numbers allow for automatic, exact evaluation of the numerical derivative of high-dimensional functions at an arbitrary point with minimal coding effort. We use dual numbers to construct tangent linear and adjoint model code for a biogeochemical ocean model and apply it to a variational (4D-Var) data assimilation system when coupled to a realistic physical ocean circulation model with existing data assimilation capabilities. The resulting data assimilation system takes modestly longer to run than its hand-coded equivalent but is considerably easier to implement and updates automatically when modifications are made to the biogeochemical model, thus making its maintenance with code changes trivial.

## Introduction

Data assimilation refers to a variety of statistical and computational techniques that rigorously constrain numerical models using observed data, with the aim of producing the most accurate estimate of the modeled state. Among many applications in the geosciences, it is applied to coupled physical-biogeochemical ocean models, often yielding large improvements in the model’s state estimates (see, e.g. [1, 2]). Like other optimization approaches, variational techniques, which are among the most widely-used data assimilation techniques, use the derivative of a cost function that measures the misfit between model output and observations, with respect to adjustable controls. Computing the derivative of a complex, high-dimensional function is not a trivial task for which a only a limited number of approaches exist [3].

One efficient approach for computing the derivative of complex nonlinear functions is automatic differentiation [4–6]: it is based on the successive differentiation of each operation in the nonlinear model code. An automatic differentiation technique that has recently gained in popularity is the use of dual numbers [7–10]. Dual numbers are an extension of real numbers similar to imaginary numbers ([11]); the result of a numerical function evaluation for a dual number contains the value of the derivative of the function (for details, see next section). That is, the exact value of a derivative of a function can be obtained without explicitly computing a derivative and with minimal coding effort in most modern programming languages.

Variational data assimilation uses the adjoint method for cost function minimization, requiring tangent linear and adjoint models which are based on the derivative of the nonlinear model [12]. If applied to the full model, dual numbers allow for the direct computation of the derivative of the cost function, avoiding the requirement for tangent linear and adjoint code altogether [9]. In many applications, however, parts of the complex data assimilation machinery are already in place and just need to be extended to accommodate a new model component. Such a case is examined in this study: we use dual numbers to build tangent linear and adjoint code for a biogeochemical model that is then coupled to and used with an existing physical circulation model, the Regional Ocean Modelling System (ROMS; [13]) which already contains variational data assimilation capabilities.

In the following, we briefly introduce dual numbers, and their implementation in the Fortran programming language, before applying them to evaluate the tangent linear and adjoint models for the North Pacific Ecosystem Model for Understanding Regional Oceanography (NEMURO) biogeochemical model [14]. We evaluate the model runtime in the results section and conclude with a discussion of these results.

## Methods

### A short introduction to dual numbers

Dual numbers augment real numbers with a dual element *ε*:

$$\begin{array}{c} d=x+y\;\varepsilon \;\;\text{with}\;\;x,y,\in R, \end{array}$$ (1)

where *x* is the real part and *y* the dual part of the dual number *d*. Dual numbers are similar to imaginary numbers in appearance and arithmetic but have the important distinction that *ε*^2^ = 0 (whereas for the imaginary element, *i*^2^ = −1). To provide an illustration of dual number arithmetic and to show how to obtain an evaluation of the derivative of a function, we use a simple example. We evaluate the function *f*(*x*) = 2(*x* − 1)^2^ + 3, with derivative *f*′(*x*) = 4(*x* − 1), for a dual number *d* = *x* + *ε y*:

$$\begin{array}{cc} f(x+y\;\varepsilon ) & =2{\left(x+y\;\varepsilon -1\right)}^{2}+3\\ & =2({\left(x-1\right)}^{2}+2\left(x-1\right)y\;\varepsilon +y^{2}\;\varepsilon^{2})+3 \end{array}$$

and because *ε*^2^ = 0

$$\begin{array}{c} =2{\left(x-1\right)}^{2}+3+4\left(x-1\right)\;y\;\varepsilon \\ =f\left(x\right)+f^{\prime }\left(x\right)\;y\;\varepsilon . \end{array}$$ (2)

That is, the real part of *f*(*x* + *y ε*) is *f* evaluated at *x*, and its dual part is *y* multiplied to the *derivative* of *f* evaluated at *x*. This result for a particular *f* generalizes to any differentiable *f*: R ↦ R which can be shown using a Taylor expansion:

$$\begin{array}{cc} f(x+y\;\varepsilon ) & =f\left(x\right)+f^{\prime }\left(x\right)\;y\;\varepsilon +\sum_{k=2}^{\infty }\frac{f^{\left(k\right)}\left(x\right)}{k!}\;y^{k}\;\varepsilon^{k}\\ & =f\left(x\right)+f^{\prime }\left(x\right)\;y\;\varepsilon +\underset{=0}{\underset{︸}{\varepsilon^{2}}}(\sum_{k=2}^{\infty }\frac{f^{\left(k\right)}\left(x\right)}{k!}\;y^{k}\;\varepsilon^{k-2})\\ & =f\left(x\right)+f^{\prime }\left(x\right)\;y\;\varepsilon . \end{array}$$ (3)

Consequently, dual numbers can be used to evaluate the exact derivative of a function without the need to explicitly derive it.

### Implicit evaluation of tangent linear and adjoint models using dual numbers

Nonlinear models $M_{\text{NL}}:R^{n}\mapsto R^{n}$ map a vector of input variables to a vector of output variables; the derivative of this mapping is the tangent linear or Jacobian matrix which is defined as

$$\begin{array}{c} M(x_{\text{NL}})=(\frac{\partial }{\partial x_{1}},\ldots ,\frac{\partial }{\partial x_{n}})M_{\text{NL}}\left(x\right){|}_{x=x_{\text{NL}}}, \end{array}$$ (4)

Here *n* is the size of the model state **x**_NL_ which, for many applications in the geosciences, can be upwards of 10^6^. The tangent linear model $M_{\text{TL}}\left(x_{\text{NL}},x_{\text{TL}}\right)=M(x_{\text{NL}})\cdot x_{\text{TL}}$ computes the matrix product of the tangent linear matrix **M**(**x**_NL_) and a vector **x**_TL_. The tangent linear model thus provides an approximation of how the output of the nonlinear model is affected by the perturbations to its input contained in **x**_TL_. The corresponding adjoint model computes the matrix product of the transpose of the tangent linear matrix and a vector, providing an approximation of how perturbations of the output of the nonlinear model relate back to its input.

Both tangent linear and adjoint models are used in the cost function minimization employed by 4D-Var and other variational data assimilation techniques (see [15, 16] and references therein). We use the above-mentioned property of dual numbers to evaluate the tangent linear model of a given nonlinear model without the need to compute a derivative explicitly. Evaluating $M_{\text{NL}}$ for a dual number yields the multivariate equivalent of Eq (3):

$$M_{\text{NL}}\left(x_{\text{NL}}+x_{\text{TL}}\varepsilon \right)=M_{\text{NL}}\left(x_{\text{NL}}\right)+\underset{M_{\text{TL}}\left(x_{\text{NL}},x_{\text{TL}}\right)}{\underset{︸}{\underset{M\left(x_{\text{NL}}\right)}{\underset{︸}{{\left(\frac{\partial }{\partial x_{1}},\ldots ,\frac{\partial }{\partial x_{n}}\right)M_{\text{NL}}\left(x\right)|}_{x=x_{\text{NL}}}}}\cdot x_{\text{TL}}}}\varepsilon .$$ (5)

And thus, the value of $M_{\text{TL}}\left(x_{\text{NL}},x_{\text{TL}}\right)$ is equal to the dual part of $M_{\text{NL}}\left(x_{\text{NL}}+x_{\text{TL}}\;\varepsilon \right)$.

The solution of the adjoint model $M_{\text{AD}}\left(x_{\text{NL}},x_{\text{AD}}\right)={M(x_{\text{NL}})}^{\text{T}}\cdot x_{\text{AD}}$ is based on the transpose of the tangent linear matrix, and dual numbers cannot be used directly to evaluate its result. The reason for this is that in a standard adjoint model approach, each variable on the right hand side of an expression maps to a separate adjoint expression with that variable on the left hand side. In general, this requires a more complex code transformation than the overloading described here. For biogeochemical models, however, we showed in [17] that we can instead obtain the result through a series of tangent linear model evaluations:

$$\begin{array}{cc} M_{\text{AD}}\left(x_{\text{NL}},x_{\text{AD}}\right) & ={M(x_{\text{NL}})}^{\text{T}}\cdot x_{\text{AD}}\\ & =\sum_{i=1}^{n}e_{i}[{\left(M(x_{\text{NL}})\cdot e_{i}\right)}^{\text{T}}\cdot x_{\text{AD}}]\\ & =\sum_{i=1}^{n}e_{i}[M_{\text{TL}}{\left(x_{\text{NL}},e_{i}\right)}^{\text{T}}\cdot x_{\text{AD}}], \end{array}$$ (6)

where **e**_*i*_ is the *i*th unit vector and **M**(**x**_NL_) ⋅ **e**_*i*_ is hence the *i*th column of **M**(**x**_NL_). That is, the tangent linear matrix is created column-by-column using *n* evaluations of the tangent linear model. Applying the relationship in Eq (5), *n* evaluations of $M_{\text{NL}}$ with dual numbers can thus be used for one evaluation of the adjoint model. Depending on the size of *n*, the large number of model evaluations may come at a considerable computational expense which we reduce in this application by exploiting typical characteristics of biogeochemical models (see below). We further replace the *n* evaluations of $M_{\text{NL}}$, using dual numbers that share the same real part, by a single evaluation using a dual number with *n* independent dual parts (see next section).

### Implementation using operator overloading

A straightforward way to implement dual number-based automatic differentiation is to define a dual number data type in the programming language of choice and to implement mathematical operators (+, −, *, /, <, etc.) and basic mathematical functions (log, exp, abs, etc.) for the new dual number data type, an approach that is referred to as operator and function *overloading*. Once the new data type and its operators and functions have been implemented, statements in the code containing mathematical operations like “*x* + 6*exp(*y*)” can be evaluated in the programming language no matter if the variables *x* and *y* therein are floating point numbers, dual numbers, or a mix thereof. Operator and function overloading is supported by most modern programming languages, including Fortran 90/95 and higher (see, e.g, [8]) and Python (see our example implementation of dual number-based tangent and adjoint models for a generic biogeochemical model [18]).

ROMS is written in Fortran 90/95, and we implemented a new dual number data type in a separate Fortran module and included corresponding overloaded operators and functions in that module. This module is not specific to ROMS and could easily be used to compute the derivative of a wide variety of Fortran functions and subroutines. Making it work with parts of the existing Fortran ROMS code, only involves importing the dual number module and redeclaring the data type of select variables as dual numbers instead of floating point numbers (which easily permits the creation of, and computation with, dual number arrays).

In the evaluation of the adjoint model (Eq (6)), *n* evaluations of $M_{\text{NL}}$ with dual numbers that share same real part can be replaced by a single evaluation of a dual number with *n* independent dual parts:

$$\begin{array}{c} d=y_{1}\;\varepsilon {}_{1}+y_{2}\;\varepsilon {}_{2}+\ldots +y_{n}\;\varepsilon {}_{n}\;\;\text{with}\;\;\varepsilon {}_{i}\;\varepsilon {}_{j}=0\;\text{for}\;i,j\in \left\{1,2,\ldots ,n\right\}, \end{array}$$ (7)

where the dual number *d* has dual parts *ε*_1_, *ε*_2_, …, *ε*_*n*_ that are independent (*ε*_*i*_ *ε*_*j*_ = 0) and thus do not interact with each other. Dual numbers with two or more independent dual parts are easy to implement on top of regular dual numbers [8, 18] and because the repeat computation of the real part is avoided, their use can lead to performance gains.

### Reducing the cost of an adjoint model evaluation

While the tangent linear model can be evaluated using one call to the nonlinear model (Eq (5)), an evaluation of the adjoint model requires *n* calls, or one call with a dual number with *n* independent dual parts, where *n* is the number of columns of the tangent linear matrix. In order to reduce the computational cost of the adjoint model, we decrease the effective size of *n* using the approach outlined in [17] which relies on two characteristics of marine biogeochemical models that lend themselves to the efficient implementation of this approach. (1) Marine biogeochemical models are naturally divided into segments, typically respresenting different biogeochemical processes. By evaluating the adjoint model for each segment individually, not all variables must be considered in Eq (6) and consequently *n* decreases for each segment. For example, the “zooplankton grazing” segment contained in most marine biogeochemical models describes the feeding of zooplankton on phytoplankton which is independent of the availability of nutrients. Hence nutrient variables do not need to be considered in the evaluation of the adjoint of the zooplankton grazing segment of $M_{\text{NL}}$. (2) Apart from two segments typically contained in biogeochemical models (namely sinking of particulate materials and light attenuation through the water column) the solution of every other segment in one grid cell is independent of other grid cells and the variables in other grid cells do not need to be considered in the evaluation of $M_{\text{AD}}$. Note that the effects of mixing and transport do depend on neighboring grid cell properties but in ROMS, the adjoint and tangent linear models for these processes are already written and require no modification for changes to the biogeochemical model. Thus, we can ignore mixing and transport. We further exclude sinking and light attenuation from the dual number-based adjoint model; both of the segments are generic in that they do not differ much between different models and existing adjoint code from the ROMS codebase was used for these two segments.

## Results

We apply the dual number-based tangent linear and adjoint model to an operational data assimilation system simulating the 3-dimensional physical circulation and biogeochemical cycling in the California current system.

### Example application

In order to illustrate the capabilities of the dual number-based data assimilation system, we use it to perform a 4D-Var assimilation cycle for a coupled physical-biogeochemical model based on ROMS (version 3.7, revision 820). The model domain encompasses the California current system from 30°N to 48°N and it extends westward from the U.S. west coast to 134°W. The horizontal model resolution is 0.1° × 0.1°; vertically, the model is split into 42 terrain-following layers. The physical circulation model is forced at its boundaries and surface (wind, solar radiation, air temperature, pressure and humidity) by output from the Coupled Ocean Atmosphere Mesoscale Prediction System (COAMPS; [19]). The biogeochemical model component, NEMURO, contains 11 biogeochemical variables, including variables for 2 phytoplankton from which model chlorophyll *a* is derived using a fixed nitrate-to-chlorophyll *a* ratio. NEMURO is part of ROMS, which also contains the physical circulation model, including the tangent linear and adjoint code for 4D-Var data assimilation with the physical model [20]. We further have access to a hand-coded version of the NEMURO tangent linear and adjoint model (presented in [2] and in which the derivative of the nonlinear model code was manually calculated and further transposed line-by-line to create the code at considerable expense of time and effort) which we use for comparison purposes here.

**Fig 1:**
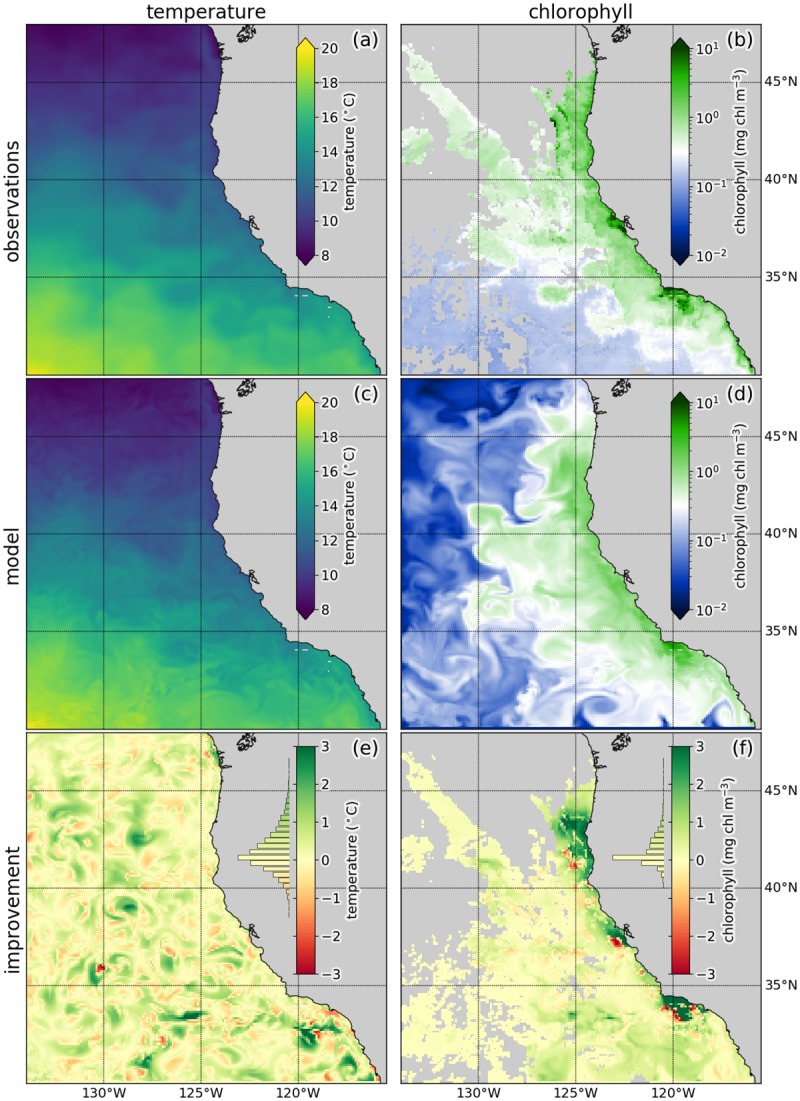
Results of a 4-day 4D-Var assimilation cycle in which observations of the physical and biogeochemical observations are assimilated jointly. Each figure shows a 4 day cycle average of surface observations (a,b), surface model state after assimilation (c,d) or model improvement at the observation locations (e,f). Here, model improvement refers to the absolute model-observation misfit before data assimilation minus the absolute misfit after data assimilation (positive numbers indicate a decrease of the misfit brought by the data assimilation, negative numbers indicate an increase). Histograms left of the color bars in e and f show the distribution of the improvement.

In the data assimilation system, in situ and remote sensing observations of physical variables (temperature, salinity, sea level anomaly, surface currents) are assimilated jointly with satellite-derived data of surface chlorophyll *a*, all obtained from publicly available data sources (listed in Table 1). We focus our description on model temperature and chlorophyll *a*, representatives of physical and biogeochemical variables for which observations are available for assimilation. Within a 4-day data assimilation cycle, a total of 144 414 observations are assimilated; because cloud cover and other atmospheric interference often inhibit the view of the optical instrument collecting the measurements used to create the chlorophyll *a* data, there are few observations of surface chlorophyll *a* in some parts of the model domain (Fig 1b). The surface temperature data used in this experiment is a higher level product that has undergone additional processing to render it gapless (Fig 1a).

**Table 1:** The data used for assimilation experiment shown in Fig 1, including the number of observations assimilated in the 4-day cycle.

observed variables	data source	number	URL
temperature (T)	OSTIA	88 180	http://ghrsst-pp.metoffice.com/ostia/
chlorophyll *a*	MODIS-Aqua	21 004	https://modis.gsfc.nasa.gov
surface currents	HFRADAR	13 368	https://cordc.ucsd.edu/projects/mapping/
sea level anomaly	AVISO	12 876	https://www.aviso.altimetry.fr
salinity (S)	Aquarius	2 656	https://podaac.jpl.nasa.gov/aquarius/
T+S	UCSD glider	4 560	https://gliders.ioos.us/data/
T+S	La Push glider	1 408	https://gliders.ioos.us/data/
T+S	Argo	362	http://www-argo.ucsd.edu/

In the assimilation, the dual number-based tangent linear and adjoint models are used in the ROMS incremental, strong constraint 4D-Var implementation in order to find adjustments to the initial conditions (that is, the state of the model variables at the beginning of the data assimilation cycle) that minimize the 4D-Var cost function. After assimilation, model temperature and its time evolution show a good agreement with the spatially dense set of temperature observations that were assimilated (Fig 1c), and only a small underestimation bias is noticeable for chlorophyll *a* in the northwest of the model domain for which little data is available within the 4-day cycle (Fig 1d). The assimilation lead to a net reduction in model-observation misfit across the 4-day cycle (Fig 1e and 1f) and the largest improvement is obtained for coastal chlorophyll *a* (for more details about the coupled physical-biogeochemical model, observations, assimilation system, and resulting improvements in state estimates, see [2] and [21]).

### Runtime

**Fig 2:**
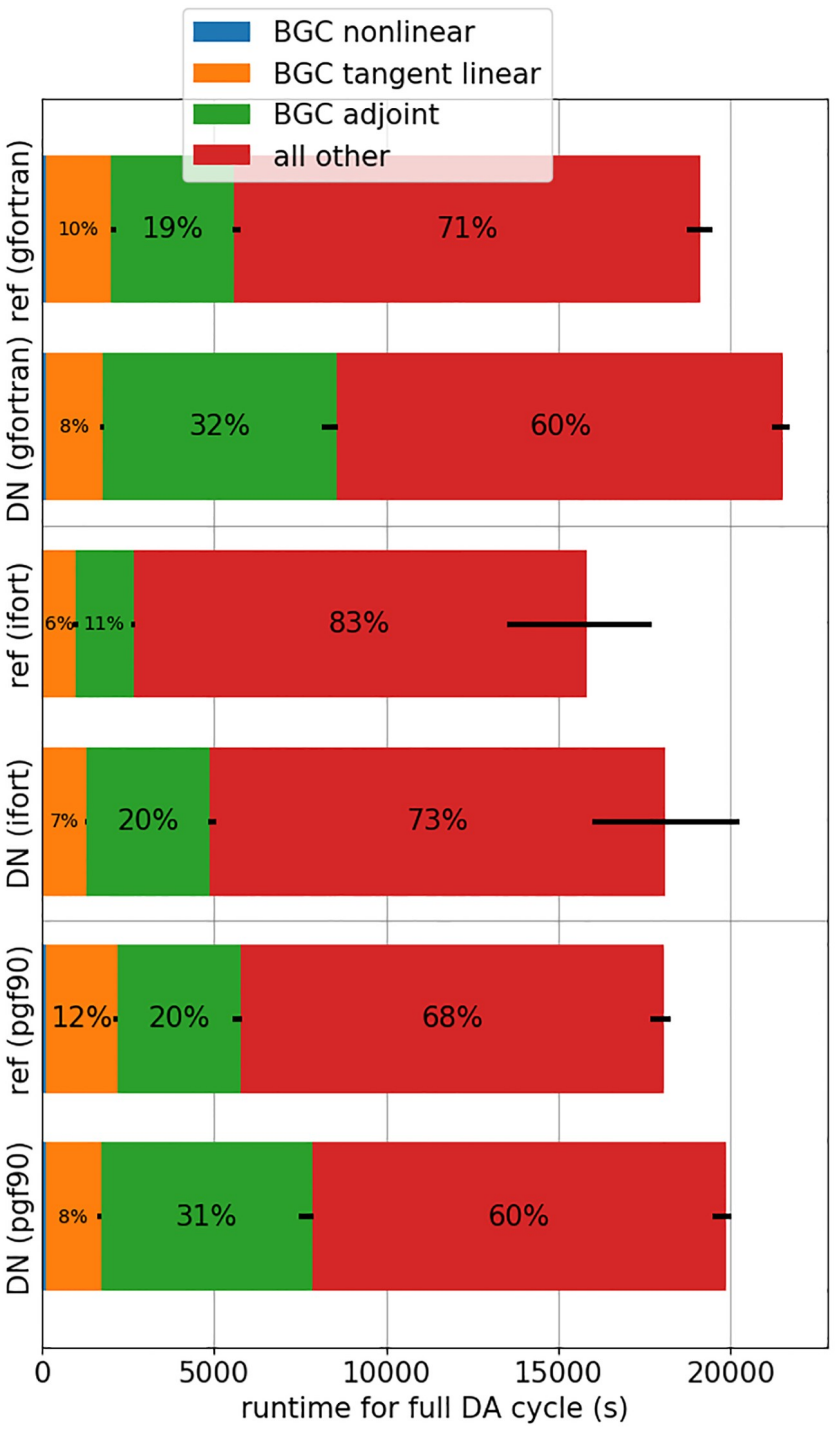
Median ROMS runtime for a full 4D-Var data assimilation cycle (10 inner, 2 outer loops) for the hand-coded reference code (ref) and the code using the dual number-based (DN) biogeochemical tangent linear and adjoint code. The length of each bar shows the median time across 30 cycles, the error bars indicate minimum and maximum runtime. Colors denote runtime of individual model parts, percentage values show relative contribution of each part to the total. We tested 3 compiler configurations. The nonlinear model evaluations, shown in blue, contribute much less than 1% to the overall runtime and are hence difficult to perceive.

After verifying that the dual number-based code replicates the results of reference code to machine precision, we focus on evaluating its runtime: for both the dual number-based and handcoded versions of the biogeochemical 4D-Var code, we perform longer data assimilation experiments consisting of 30 4-day cycles in which physical and satellite chlorophyll *a* observations are again assimilated jointly. In a previous study, we noted a strong dependence of 4D-Var runtime on the Fortran compiler used to create the ROMS executable [17]. For this reason, we base our experiments on 3 different compiler configurations which use 3 Fortran compilers available to us. For each configuration, we compare the median runtime of 30 assimilation cycles for the dual number-based code and the hand-coded reference. Again, our results vary considerably among the different configurations (Fig 2). For the tangent linear model, the dual number-based code shows a decreased median runtime for two of our compiler configurations and an increase for the third (changes of −22%, −12% and +34%, respectively; yellow bars in Fig 2). The dual number-based adjoint code utilizes dual numbers with more than one dual part, which increases its runtime relative to the reference code in all of our configurations. We see an increase in median runtime between 71% and 113% (green bars in Fig 2). Overall, the dual number-based code is slightly slower than the hand-coded version: it is associated with an average increase in median runtime between 10% and 15% for a full data assimilation cycle in comparison to the reference simulation in our application.

## Discussion & conclusions

The computation of derivatives of complex, high-dimensional functions is fundamental to a wide variety of scientific applications, from statistical inference [22] to weather forecasting [16]. Dual numbers offer an automated way to compute exact derivatives of these functions with minimal coding effort. We presented an application of dual numbers to an operational ocean data assimilation system in which observations of the physical and biological ocean state are used to improve estimates of ocean currents, phytoplankton biomass and primary productivity, among other ocean variables. In our application, dual numbers are used to create exact tangent linear and adjoint code for the biological ocean component.

One main advantages of the use of dual numbers is its ease of implementation and flexibility of the code: because the code for data assimilation is based on nonlinear model evaluations (with dual numbers), changes to the biogeochemical ocean model do not require any further update of the data assimilation system. This characteristic requires not only the dual number-based implementation of the tangent linear model, but also of the more complex adjoint model; a novel aspect examined in this study is the construction of an adjoint model based on a single evaluation of the nonlinear model with multiple dual parts. Our approach implicitly reconstructs the tangent linear matrix column-by-column, which is associated with a higher computational cost compared to an evaluation of the tangent linear model. By exploiting common features of biogeochemical model, we can reduce this cost significantly, though an evaluation of the dual number-based adjoint model remains more costly than its hand-coded reference version, resulting in a 10%-15% increase in runtime for our full data assimilation system. (Options to speed up the code, such as storing the tangent linear matrices computed in the tangent linear model and reusing them in the adjoint model [17] or basing the full data assimilation system on dual numbers as in [9] are associated with a much larger coding effort and are not examined here.) We maintain that the ease of implementation and reduced risk to incorporate errors into the code outweigh this disadvantage for many applications.

In summary, we presented an application of dual number-based automatic differentiation to obtain exact tangent linear and adjoint code for use in a realistic, operational coupled physical-biogeochemical data assimilation system for the California Current. The benefit of this approach relative to the standard method of separately developing tangent linear and adjoint models is the substantially reduced initial development cost and near-complete elimination of effort to maintain consistency between the different models when the code changes are introduced to the nonlinear model. Its disadvantage is an increase in runtime, mainly associated with dual number-based adjoint model (for the NEMURO model used here, the increase is less than 15%). This method is therefore particularly well-suited for applications in which simulated processes are primarily local to grid cells, which helps to reduce the runtime of an adjoint model evaluation. These applications include biogeochemical models and models of sea ice or atmospheric chemistry, though the computational efficiency of the described approach will depend on the specific model.

## References

[1] Edwards CA, Moore AM, Hoteit I, Cornuelle BD. Regional Ocean Data Assimilation. Annual Review of Marine Science. 2015;7(1):21–42. 10.1146/annurev-marine-010814-015821 25103331

[2] Mattern JP, Song H, Edwards CA, Moore AM, Fiechter J. Data assimilation of physical and chlorophyll a observations in the California Current System using two biogeochemical models. Ocean Modelling. 2017;109:55–71. 10.1016/j.ocemod.2016.12.002

[3] Martins JRRA, Hwang JT. Review and Unification of Methods for Computing Derivatives of Multidisciplinary Computational Models. AIAA Journal. 2013;51(11):2582–2599. 10.2514/1.J052184

[4] Griewank A, Walther A. Evaluating derivatives: principles and techniques of algorithmic differentiation. Vol. 105 Siam, 2008.

[5] Giering R, Kaminski T. Recipes for adjoint code construction. ACM Transactions on Mathematical Software. 1998;24(4):437–474. 10.1145/293686.293695

[6] Giering R. Tangent linear and adjoint biogeochemical models. Inverse methods in global biogeochemical cycles. Vol. 114; 2000 p. 33–48. 10.1029/GM114p0033

[7] Leuck H, Nagel HH. Automatic differentiation facilitates OF-integration into steering-angle-based road vehicle tracking. In: Proceedings. 1999 IEEE Computer Society Conference on Computer Vision and Pattern Recognition (Cat. No PR00149). IEEE Comput. Soc; 1999. p. 360–365.

[8] Yu W, Blair M. DNAD, a simple tool for automatic differentiation of Fortran codes using dual numbers. Computer Physics Communications. 2013;184(5):1446–1452. 10.1016/j.cpc.2012.12.025

[9] Wang G, Cao X, Cai X, Sun J, Li X, Wang H. A new data assimilation method for high-dimensional models. PLOS ONE. 2018;13(2):1–15. 10.1371/journal.pone.0191714 PMC5805242 29420553

[10] Orr JC, Epitalon JM, Dickson AG, Gattuso JP. Routine uncertainty propagation for the marine carbon dioxide system. Marine Chemistry. 2018;207:84–107. 10.1016/j.marchem.2018.10.006

[11] Harkin AA, Harkin JB. Geometry of Generalized Complex Numbers. Mathematics Magazine. 2004;77(2):118–129. 10.1080/0025570X.2004.11953236

[12] Le Dimet F, Talagrand O. Variational algorithms for analysis and assimilation of meteorological observations: theoretical aspects. Tellus A. 1986;38A(2):97–110. 10.3402/tellusa.v38i2.11706

[13] Haidvogel DB, Arango HG, Budgell WP, Cornuelle BD, Curchitser EN, Di Lorenzo E, et al Ocean forecasting in terrain-following coordinates: Formulation and skill assessment of the Regional Ocean Modeling System. Journal of Computational Physics. 2008;227:3595–3624. 10.1016/j.jcp.2007.06.016

[14] Kishi MJ, Kashiwai M, Ware DM, Megrey BA, Eslinger DL, Werner FE, et al NEMURO—a lower trophic level model for the North Pacific marine ecosystem. Ecological Modelling. 2007;202(1-2):12–25. 10.1016/j.ecolmodel.2006.08.021

[15] Thépaut JN, Courtier P. Four-dimensional variational data assimilation using the adjoint of a multilevel primitive-equation model. Quarterly Journal of the Royal Meteorological Society. 1991;117(502):1225–1254. 10.1002/qj.49711750206

[16] Courtier P, Thépaut JN, Hollingsworth A. A strategy for operational implementation of 4D-Var, using an incremental approach. Quarterly Journal of the Royal Meteorological Society. 1994;120(519):1367–1387. 10.1002/qj.49712051912

[17] Mattern JP, Edwards CA. A simple finite difference-based approximation for biogeochemical tangent linear and adjoint models. Journal of Geophysical Research: Oceans. 2019;124(1):4–26.

[18] Mattern JP. GitHub repository with dual number example implementation. GitHub:https://github.com/jpmattern/dualnum.

[19] Doyle JD, Jiang Q, Chao Y, Farrara J. High-resolution real-time modeling of the marine atmospheric boundary layer in support of the AOSN-II field campaign. Deep-Sea Research Part II: Topical Studies in Oceanography. 2009;56(3-5):87–99. 10.1016/j.dsr2.2008.08.009

[20] Moore AM, Arango HG, Broquet G, Powell BS, Weaver AT, Zavala-Garay J. The Regional Ocean Modeling System (ROMS) 4-dimensional variational data assimilation systems. Part I—System overview and formulation. Progress in Oceanography. 2011;91(1):34–49. 10.1016/j.pocean.2011.05.004

[21] Mattern JP, Edwards CA, Moore AM. Improving Variational Data Assimilation through Background and Observation Error Adjustments. Monthly Weather Review. 2018;146(2):485–501. 10.1175/MWR-D-17-0263.1

[22] Hoffman MD, Gelman A. The No-U-Turn Sampler: Adaptively Setting Path Lengths in Hamiltonian Monte Carlo. Journal of Machine Learning Research. 2014;15:1593–1623. ArXiv:1111.4246.

